# Additive QTLs on three chromosomes control flowering time in woodland strawberry (*Fragaria vesca* L.)

**DOI:** 10.1038/hortres.2017.20

**Published:** 2017-05-24

**Authors:** Samia Samad, Takeshi Kurokura, Elli Koskela, Tuomas Toivainen, Vipul Patel, Katriina Mouhu, Daniel James Sargent, Timo Hytönen

**Affiliations:** 1 Department of Agricultural Sciences, Viikki Plant Science Centre, University of Helsinki, 00014 Helsinki, Finland; 2 Fondazione Edmund Mach, Research and Innovation Centre, San Michele All'adige, 38010 TN, Italy; 3 Faculty of Agriculture, Utsunomiya University, Tochigi, 321-8505, Japan; 4 Department of Plant Developmental Biology, Max Planck Institute for Plant Breeding Research, 50829 Cologne, Germany; 5 Driscoll’s Genetics Limited, East Malling Enterprise Centre, East Malling, Kent ME19 6BJ, UK; 6 Department of Biosciences, Viikki Plant Science Centre, University of Helsinki, 00014 Helsinki, Finland

## Abstract

Flowering time is an important trait that affects survival, reproduction and yield in both wild and cultivated plants. Therefore, many studies have focused on the identification of flowering time quantitative trait locus (QTLs) in different crops, and molecular control of this trait has been extensively investigated in model species. Here we report the mapping of QTLs for flowering time and vegetative traits in a large woodland strawberry mapping population that was phenotyped both under field conditions and in a greenhouse after flower induction in the field. The greenhouse experiment revealed additive QTLs in three linkage groups (LG), two on both LG4 and LG7, and one on LG6 that explain about half of the flowering time variance in the population. Three of the QTLs were newly identified in this study, and one co-localized with the previously characterized *FvTFL1* gene. An additional strong QTL corresponding to previously mapped *PFRU* was detected in both field and greenhouse experiments indicating that gene(s) in this locus can control the timing of flowering in different environments in addition to the duration of flowering and axillary bud differentiation to runners and branch crowns. Several putative flowering time genes were identified in these QTL regions that await functional validation. Our results indicate that a few major QTLs may control flowering time and axillary bud differentiation in strawberries. We suggest that the identification of causal genes in the diploid strawberry may enable fine tuning of flowering time and vegetative growth in the closely related octoploid cultivated strawberry.

## Introduction

Synchronizing floral development with local climatic conditions is important for crop and wild plants because of its significant impact on reproduction, yield and ultimately survival. The genetic control of flowering time is thus of immense significance. As a result, numerous mapping studies have focused on the identification of flowering time QTLs in different crops, and a detailed molecular understanding of flowering time regulation in model plants has facilitated the identification of causal genes.^1–4^ In the cultivated strawberry, one of the most economically important berry crops throughout the world, flowering time is a major breeding target because of year-round demand for fresh berries. To extend the cropping season, breeders are developing early and late flowering cultivars as well as everbearing (EB; also called day-neutral) cultivars, which display a continuous flowering habit.^5–8^

In seasonal flowering ‘Junebearing’ strawberries, flower induction occurs in autumn, and the timing of the induction correlates with flowering time in the following season.^9^ Both short days (SD) and cool temperatures can induce flowering. Typically, flower induction occurs independently of day-length at temperatures below ~13 °C, whereas SDs are required at intermediate temperatures, and at temperatures above 20–24 °C floral induction is inhibited.^10–13^ There is however significant variation in critical temperature limits between cultivars,^10,14^ and some cultivars possess an obligatory SD requirement for flower induction even under cool temperatures.^15^ In contrast to seasonal flowering strawberries, EB cultivars flower earlier under long days (LD) than SDs.^16,17^ Both seasonal flowering and EB habits are also found in the woodland strawberry.^18^

As the cultivated strawberry is a complex allo-octoploid (2*n*=8*x*=56), the woodland strawberry (*F. vesca*; 2*n*=2*x*=14) and other diploid species have been used as a genetically facile surrogate system for genetic investigation. Several genetic linkage maps have been developed for diploid strawberry species. Initially saturated maps were developed using large numbers of microsatellite (SSR) and other PCR-based markers,^19–25^ and the *Fragaria* SSR-based reference linkage map was used to anchor the *F. vesca* ‘Hawaii-4’ genome sequence.^26^ More recently, high-throughput genotyping has been employed using various platforms to produce dense SNP-based linkage maps that have enabled more precise sequence scaffold anchoring and orientation,^27^ whereas the most comprehensive linkage map for diploid *Fragaria* to date is that of *F. iinumae* produced using the *Fragaria* Axiom SNP genotyping array (Axiom IStraw90, Affymetrix, Santa Clara, CA, USA), supplemented with SNPs scored using genotyping-by-sequencing (GBS).^28,29^ The linkage maps have been used to map a number of genetic loci in diploid strawberry species, including fruit color, runnering, leaf color, and seasonal flowering,^20,30–32^ and represent a powerful tool for the genetic dissection of traits of economic importance in the cultivated strawberry.

Previous genetic mapping studies have shown that the gene causing seasonal flowering habit in woodland strawberry is located on linkage group 6 (LG6).^21,31^ This gene has been recently shown to encode a strong repressor of flowering, TERMINAL FLOWER 1 (FvTFL1), and a 2-bp deletion in the first exon of *FvTFL1* causes expression of the EB phenotype in woodland strawberry.^32^ Furthermore, homologs of FvTFL1 have been found to repress flowering in several rosaceous species suggesting the conservation of the genetic mechanism of flowering time regulation.^33–36^ Only a few other flowering time genes have been functionally characterized in woodland strawberry. Using genetic transformation, recent studies have revealed how homologs of *FLOWERING LOCUS T* (*FvFT1*) and *SUPPRESSOR OF THE OVEREXPRESSION OF CONSTANS1* (*FvSOC1*) mediate photoperiod and temperature signals and how they are integrated to control flower induction.^32,37–39^ In the seasonal flowering woodland strawberry, cool temperatures below 13 °C cause photoperiod-independent downregulation of *FvTFL1* by an unknown mechanism leading to flower induction, whereas at higher temperatures, photoperiod controls flowering through *FvTFL1*. Under LD, leaf-expressed FvFT1 activates *FvSOC1* in the shoot apex leading to the upregulation of *FvTFL1* and the maintenance of a vegetative stage, whereas under SD, this *FvFT1-FvSOC1-FvTFL1* pathway is silenced and flower induction occurs. However, at temperatures above 20 °C *FvTFL1* is highly upregulated by an unknown activator that functions independently of FvSOC1, and plants remain vegetative.^37,39^ This model contrasts with the mechanism in *Arabidopsis* in which both FT and SOC1 function as floral activators.^1^ However, FvFT1 is a strong floral activator in EB woodland strawberries that are lacking functional *FvTFL1*.^32,38^

The genetic control of the EB habit has also been studied in the cultivated strawberry. Weebadde *et al.*^5^ suggested the presence of several QTLs with significant genotype×environment interaction,^5^ whereas other studies indicated that a single dominant locus causes continuous flowering.^40,41^ The presence of a single QTL was supported by recent genetic mapping studies by two groups,^6,8^ who found a major dominant QTL on LG4 in two independent crossing populations.^6,8^ Moreover, Honjo *et al.*^42^ demonstrated that the EB trait is controlled by the same gene in the EB parents of these populations.^42^ In addition to the EB trait, this locus called *PFRU* also controls the production of runners, which are long shoots that enable the efficient clonal reproduction of the species.^6^ Although studies indicate that different genes cause the EB phenotype in the woodland and cultivated strawberries, a recent study has shown that *TFL1* homologs encode major floral repressors in both species, and that the silencing of this gene also causes the EB habit in cultivated strawberry.^43^ Moreover, the regulation of *FaTFL1* correlates with flower induction in different seasonal flowering cultivars.^43,44^

As strawberries form terminal inflorescences from the apical meristems of the crowns, the number of inflorescences, and consequently the yield potential, depends on the number of side shoots called branch crowns that are produced.^45–48^ SD conditions promote the differentiation of strawberry axillary buds to branch crowns, whereas under LDs axillary buds typically differentiate to runners.^10,14,45^ Changes in gibberellin biosynthesis and signaling are known to mediate the photoperiodic differentiation of axillary buds.^49^ This differentiation is not understood at the molecular level, although runnerless mutants are known in woodland strawberry.^18^

Here, we aimed to identify novel genetic loci controlling flowering time and axillary bud differentiation using the large woodland strawberry F2 mapping population produced by Koskela *et al.*^32^ We demonstrate that five QTLs with additive effects explain about half of the flowering time variance found in the population. One of the QTLs co-localized with the previously identified *FvTFL1* and another with *PFRU*, whereas the others were newly identified in this study. In addition, we report the mapping of two QTLs that affect the number of branch crowns and runners in our mapping population.

## Materials and methods

### Mapping population and phenotyping

Selected lines from a F2 population derived from the cross *F. vesca f. semperflorens* ‘Hawaii-4’ (H4)×*F. vesca* subsp*. vesca* (FV), (denoted H4×FV),^32^ were used in this study. The population consisted of 735 seasonal flowering plants that were planted in an experimental field at the University of Helsinki at the beginning of August 2009. In the spring 2010, flowering time was observed in the field. On the basis of these observations and previously scored genotypes for the *FvTFL1* gene,^32^ 335 seasonal flowering lines containing one or two functional *FvTFL1* alleles were selected for flowering time observations. At the beginning of August 2010, four clonal daughter plants per line were potted into 6 cm square plastic pots filled with peat moss (Kekkilä, Finland). Plants were kept under natural environmental conditions and periodically supplemented with fertilizer (NPK 17-4-25; Kekkilä) applications until 13 December 2010. Then, the plants were moved into a greenhouse and repotted into 8×8 cm plastic pots and arranged according to completely randomized design. The greenhouse temperature was set to 18 °C and plants were kept under an 18 h photoperiod illuminated with high pressure sodium lamps (Airam 400 W, Kerava, Finland) providing a light intensity of 100 μmol m^−2^ s^−1^. Plants were automatically watered with the fertilizer solution. The date of first flower of all genotypes was recorded and flowering time was calculated as days after the anthesis of the earliest flowering F2 line. Runners were removed and counted regularly in the greenhouse, and the total number of runners produced per plant before 8 March 2011 was calculated. In addition, the number of branch crowns was observed on the same date. On the basis of the greenhouse phenotypic data, 32 F2 lines with extreme flowering time values (16 early and 16 late flowering lines) were selected for growth chamber experiment. Four clones of each F2 line were rooted in 8×8 cm plastic pots and plants were subjected to 12 h short-day treatment (AP67 LED lamps; 200 μmol m^−2^ s^−1^; Valoya, Finland) at 11 °C for 6 weeks followed by flowering time observations in the greenhouse as described above. In the greenhouse and growth chamber experiments, mean phenotypic values of four replicates were used for QTL mapping.

### DNA extraction and quantification

DNA was extracted from young leaves of the parents ‘H4’ and ‘FV’, along with the F1 and 335 F2 seedlings of the progeny, using a modified version of the CTAB method.^31^ The DNA was quantified using a Nanodrop spectrophotometer (Thermo Fisher Scientific, Waltham, MA, USA) and was diluted 1:100 (5–10 ng μl^−1^) for use in PCR. For genome resequencing an additional purification step was carried out. NaCl was added at a final concentration of 0.2 m, and DNA was precipitated by adding 2/3 volumes of cold isopropanol. After centrifugation, the pellet was washed with 100 μl of 70% ethanol and re-suspended in 50 μl of TE.

### Amplification and scoring of genetic markers

A total of 73 published SSR primer pairs (Supplementary File 1) labeled on the forward primer with either 6-FAM or HEX fluorescent dyes^19–22,25,50–52^ were screened for polymorphism in the parental and F1 lines using the ‘Type-it’ PCR mastermix (Qiagen, Hilden, Germany), following the PCR protocol reported in Sargent *et al.*^53^ In addition, 12 SSR primer pairs were tested using the fluorescent labeling method described by Schuelke^24^ (Supplementary File 1). Samples were diluted 1:75, separated by capillary electrophoresis (Foster City, CA, USA) on a genetic analyzer (ABI 3100, ABI 3130 XL or ABI 3700, Applied Biosystems), and the resultant data were collected and analyzed using GeneMapper or Peak Scanner 2 (Applied Biosystems).

SNPs were identified in the parental genome sequences and the temperature switch PCR (TSP) protocol^23^ was used to develop assays for 49 SNP markers within flowering time QTL regions identified in this investigation. In brief, two sets of primers, a locus specific (LS) set and a nested-locus specific (NLS) set were designed per SNP using Primer3 v.0.40 (ref. 54) and NetPrimer (http://www.premierbiosoft.com/netprimer/netprlaunch/netprlaunch.html), respectively. The LS primers were designed to have a Tm between 60–65 °C (optimal 63 °C) and a product size >400 bp, whereas the NLS primers were designed to have a *T*_m_ of 43–47 °C (optimal 45 °C) and were extended using a non-complementary 5′ tail region that increased the overall primer *T*_m_ to 52–55 °C (optimal 53 °C).^23^ PCR was performed as described previously.^23^ The primer concentrations and the numbers of PCR cycles were adjusted for each primer individually to optimize marker amplification for ease of scoring. Products were visualized over UV light following electrophoresis through a 1.5% (w/v) TAE agarose gel containing gel red (Applied Bioprobes, Rockville, MD, USA) at a constant voltage of 100 V for 2–3 h, depending on the sizes of the fragments produced. The TSP loci were named UH, followed by the chromosome number and finally a unique primer pair reference number (Supplementary File 1).

### Genotyping-by-sequencing

DNA samples (30–100 ng μl^−1^) of 188 seedlings selected to represent the full seedling population genotypically (based on the SSR and TSP data) and phenotypically, were sent to the Cornell University where genotyping-by-sequencing (GBS) was carried out according to Elshire *et al.*^55^ GBS libraries were sequenced using the Illumina HiSeq2500 platform (San Diego, CA, USA). GBS tags with a minimum number of three reads and unique alignment to the reference genome (https://www.rosaceae.org/species/fragaria_vesca/genome_v2.0.a1)^27^ were identified, and from them SNPs were called by Genomic Diversity Facility in Cornell using the GBS-pipeline^56^ implemented in the TASSEL 3.0 software application.^57^ To remove genotyping errors, several filtering steps were performed for each polymorphic site using vcftools v. 0.1.12b.^58^ A site was accepted if genotype calling rate was over 90% per site, the minor allele frequency was >0.1, and the proportion of heterozygote genotypes fit to the Hardy–Weinberg expectations (2pq, *P*<0.01). Missing data was imputed with BEAGLE4 (ref. 59) and the TASSEL5 was used to produce ABH file with 4672 SNPs. Following an initial round of linkage mapping (described below), a second filtering and manual imputation step was performed using the rationale previously reported by Ward *et al*.^60^ This second imputation step removed suspected false homozygous calls that created multiple double-recombination events, inflating mapping distances and confounding local marker ordering.

### Heritability, linkage mapping and QTL analysis

The broad sense heritability for flowering time and numbers of runners and branch crowns were calculated in a population of 188 individuals according the formula *H*^2^=*σ*^2^*g*/(*σ*^2^*g*+*σ*^2^*e*), where *σ*^2^*g*=(MS(genotype)−MS(environment))/*r* and *σ*^2^*e*=MS(environment). MS(genotype) and MS(environment) were obtained from analysis of variance for completely randomized design as the mean sums of squares for genotype and residual error, respectively, and *r* was the effective number of replicates (three to four per genotype). Estimation of these variance components were performed by REML as implemented in PROC VARCOMP procedure in SAS/STAT software, version 9.4 (Copyright 2013, SAS Institute, Cary, NC, USA). Linkage mapping was performed using JOINMAP 4.1 (Kyazma, Wageningen, The Netherlands)^61^ using maximum likelihood and the default mapping criteria. The maps presented were illustrated using MapChart 2.2.^62^ The significance of association between individual markers on the H4×FV linkage map and the phenotypes was calculated with linear regression using MapQTL 6.0 (Kyazma).^63^ QTLs were identified employing interval mapping with a step size of 1 cM, considering a maximum of five neighboring markers. The genome-wide log of odds (LOD) threshold was determined over 10 000 permutations and the most significant markers were then used as co-factors for restricted multiple QTL mapping (rMQM) with a step size of 1 cM. Linkage mapping and QTL analyses were performed on 335 individuals using SSR and TSP markers and on 186 individuals using all marked data.

### Genome resequencing and the identification of single-nucleotide polymorphisms between the ‘H4’ and ‘FV’ genomes

The genome of the ‘FV’ parent was re-sequenced at DNA Sequencing and Genomics Laboratory, Institute of Biotechnology, University of Helsinki, Finland. Briefly, DNA was sheared using a Bioruptor NGS sonicator (Diagenode, Denville, NJ, USA) and the obtained fragments were end-repaired and A-tailed, and truncated Illumina Y-adapters were ligated. In a PCR step (20 cycles), full-length P5 and indexed P7 adapters were introduced using KAPA Hifi DNA Polymerase (KAPA Biosystems, Wilmington, MA, USA). The obtained libraries were purified and size selected using AMPure XP beads (Beckman Coulter, Brea, CA, USA). The obtained final libraries were paired-end (300 bp+300 bp) sequenced on a MiSeq Sequencer (Illumina, San Diego, CA, USA). Adapter sequences were removed from paired-end reads with cutadapt 1.8.1.,^64^ and reads were aligned against the strawberry reference genome v2.0.a1 with bwa-mem^65^ using default settings. After the alignment, samtools 1.3 (refs 66,67) was used for sorting, indexing, filtering and removing PCR duplicates. Reads with mapping quality of <25 were discarded. After filtering, average coverage was 14.8, and 91.5% of the reference genome was covered. Genetic variants including SNPs and indels were called with bcftools after calculating genotype likelihoods with samtools mpileup function. To ensure high quality variants, additional filtering was conducted with vcftools,^58^ and only sites having at least 10-fold coverage and <50-fold coverage were accepted for further analysis. Genomic regions spanning flowering time QTL were identified from the genomic positions of flanking SSR markers mapped in the full mapping progeny, within which polymorphic SNPs were identified for subsequent marker development.

### Identification of candidate genes in the QTL regions

To identify candidate genes for the QTLs, predicted genes located between the markers flanking the most significant markers were BLAST searched against the RefSeq or UniRef90 protein database using the blastx algorithm in Blast2GO^68^ or blast+ (2.4.0). Query sequences longer than 8000 bp were restricted to the first 8000 bp for Blast2GO as this is the maximum length accepted by the software. Homologs of known flowering time genes were selected as candidate genes. Parental genetic variation in the coding regions of candidate genes were retrieved in the Illumina whole genome resequencing data using vcftools,^58^ and translated amino acid sequences were compared to reveal non-synonymous variation in the coding regions.

## Results

### Segregation of flowering time and vegetative traits in H4×FV mapping population

**Figure 1:**
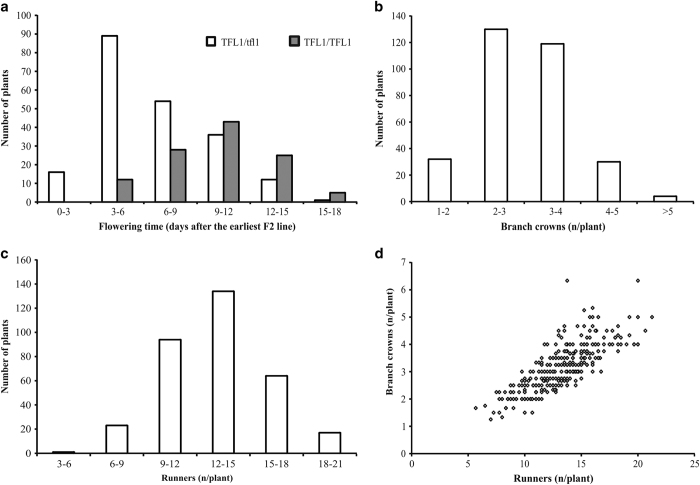
Segregation of flowering time and vegetative traits in H4×FV mapping population. Segregation of flowering time (**a**), the number of branch crowns (**b**) and runners (**c**), and correlation between the number of branch crowns and runners (**d**) are shown. Flowering time data is shown separately for plants homozygote or heterozygote for functional *FvTFL1* alleles (FV alleles). Four clones of each of 335 F2 plants were phenotyped and mean values were used for analyses. Standard deviations of clones of each F2 line varied between 0.0 and 4.0 for flowering time, 0.0 and 2.1 for branch crowns, and 0.5 and 7.6 for runners.

Initial field observations revealed over one month variation in flowering time among the 735 seasonal flowering F2 lines of H4×FV mapping population in Helsinki in summer 2010 (Supplementary Figure 1). The first plants started to flower at week 20, and almost 70% of the plants flowered by week 22. However, many plants flowered significantly later, and about 5% of plants flowered at week 25 or later. In autumn 2010, 335 F2 selected lines were subjected to natural autumn conditions followed by growth observations in a greenhouse. Again, significant variation in flowering time was observed (Figure 1a). On average, these plants started to flower 39 days after the transfer to the greenhouse, and an 18-day variation in flowering time was observed. The broad sense heritability for flowering time was *H*^2^=0.61. Greenhouse flowering data correlated positively, but weakly with field data (*r*=0.39). Flowering time of the 16 earliest and the 16 latest F2 lines were further tested in a growth chamber. In this experiment, over two week variation in flowering time and clear positive correlation with greenhouse flowering data (*r*=0.71) was observed (Supplementary Figure 2).

Variation was also observed in the vegetative development of the F2 lines. By the end of the experiment, most F2 lines produced 2–4 branch crowns per plant. However, a significant number of genotypes produced an average of less than two, or more than four branch crowns (Figure 1b). The total number of runners produced per genotype varied between 5 and 22 (Figure 1c). The broad sense heritability (*H*^2^) was 0.48 and 0.41 for branch crowns and runners, respectively. The number of runners was positively correlated with the number of branch crowns (*r*=0.785; Figure 1d). However, no correlation was found between flowering time and the number of branch crowns or runners.

### Marker development and linkage map construction

**Figure 2:**
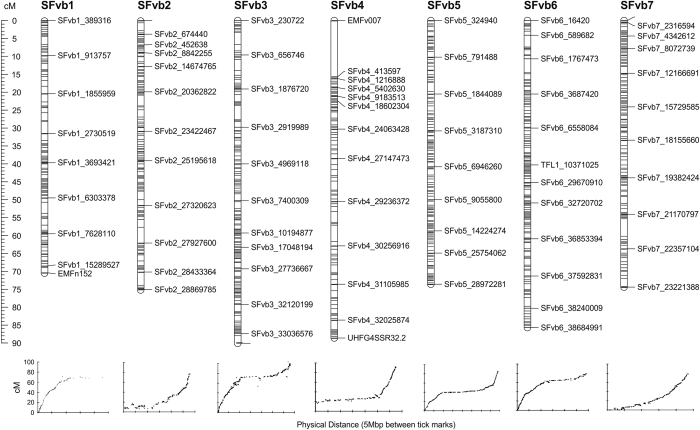
Linkage map of 186 F2 progeny of the H4×FV cross comprising of 2395 molecular markers. The numbers at the top denote the chromosome number and the genetic distances are in centiMorgan (cM, scale in the left). In the small panels, all markers of each chromosome are plotted according to their position in the genome v.2.0 (*x*-axis) and in the genetic map (*y*-axis).

Of the 73 reported SSR and 49 TSP markers screened (Supplementary File 1), 47 and 13 markers, respectively, were polymorphic between the ‘H4’ and ‘FV’ genotypes and segregated in the progeny. Following linkage analysis on 335 F2 lines, these 60 markers coalesced into seven discrete linkage groups spanning the majority of the *Fragaria* genome (Supplementary Figure 3). To increase mapping resolution, GBS was carried out in 188 F2 seedlings. Out of 271 473 572 barcoded Illumina reads, 2 391 856 GBS tags with a minimum coverage of three were found, and 1 755 780 tags (73.4%) were uniquely aligned on the H4 reference genome. Initial SNP calling revealed 133 930 SNPs, but after Hardy–Weinberg filtering and removal of SNPs that had more than 10% missing data and a minor allele frequency below 0.1, an ABH file of 4672 SNPs was retained. Following initial mapping, imputation and filtering, 2395 loci in 186 F2 lines, comprising 53 SSR/TSP markers and 2342 GBS-derived SNP markers coalesced into a linkage map of the seven expected linkage groups that covered a total genetic distance of 558 cM (Figure 2). Plotting genetic distances against the physical positions of the markers on the *Fvb* genome sequence assembly demonstrated that the genetic map was predominantly co-linear with the genome sequence (Figure 2) and thus marker placement on the map could be considered generally reliable.

### Flowering time QTLs in greenhouse and field experiments

**Figure 3:**
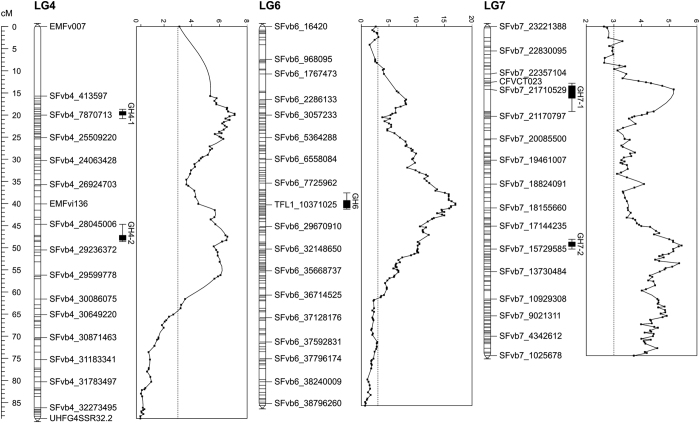
Flowering time QTLs in a greenhouse experiment. QTLs for flowering time were detected on chromosomes 4, 6, and 7. The genetic map (left) and LOD scores (right) are shown for each chromosome. Bars denote areas with the highest LOD scores (QTL regions). Field-grown plants were moved to the greenhouse in the middle of December 2011 for flowering time observations. Four clones of each of 186 F2 plants were phenotyped and mean values were used for the QTL analysis.

**Figure 4:**
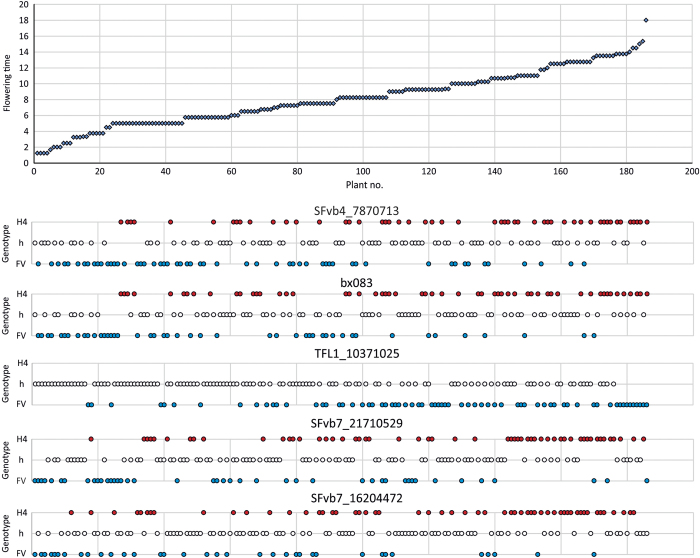
The effect of different haplotype combinations on flowering time. Flowering time of each 186 F2 line and their haplotypes on five QTL regions are shown. Haplotypes were inferred from the marker with the highest LOD score for each QTL region. H4 and FV denote parental genotypes (H4=*F. vesca f. semperflorens* Hawaii-4, FV=*F. vesca *spp.* vesca*). h=heterozygote. Flowering time is shown as days after the earliest flowering line.

QTL analysis was first carried out using greenhouse flowering time data on the progeny of 335 seedlings and segregation data for 60 genetic loci. This analysis revealed four significant QTLs, one on both LG6 and LG7, and two on LG4 (Supplementary Figure 4). QTL analysis was also performed in growth chamber flowering time data using 32 F2 lines that were selected based on their extreme flowering time phenotypes in the greenhouse experiment. Using these lines, the same four QTLs with a LOD value above 3 were detected in both greenhouse and growth chamber flowering time data (Table 1). To increase the mapping resolution, we performed QTL analysis using the additional GBS based markers on selected 186 seedlings including individuals that exhibited informative recombination close to the QTL regions mapped in a larger progeny. This high-resolution mapping identified one additional QTL peak on LG7 and narrowed down the other QTL peaks (Figure 3). The peak of the LOD of the QTL on LG6 corresponded to the position of the *FvTFL1* gene previously identified by Koskela *et al.*^32^ (Figure 3).^32^ This QTL explained 17% of the observed variance; plants homozygous for ‘FV’ alleles of *FvTFL1* flowered later than heterozygous lines (Table 2). The peaks of the LOD for two significant loci on LG4 were associated with markers SFvb4_7853414 and bx083, both explaining about 15% of the observed variance. In both cases, the allele from the ‘FV’ parent promoted flowering. The peaks of the LOD of the significant QTLs on LG7 were associated with markers SFvb7_16204472 and SFvb7_21710529 that explained over 12% of the observed variance each, with the allele of the ‘FV’ parent promoting earlier flowering. The analysis of different haplotype combinations indicated that QTLs on all three LGs had additive effects on flowering time (Figure 4; Table 2). Plants with ‘FV’ markers in both LG4 and LG7 flowered first, whereas the latest plants typically had one or two ‘H4’ alleles in both LGs. In addition, lines containing two functional *FvTFL1* alleles tended to flower later than heterozygous lines, regardless of LG4 and LG7 marker genotypes.

**Figure 5:**
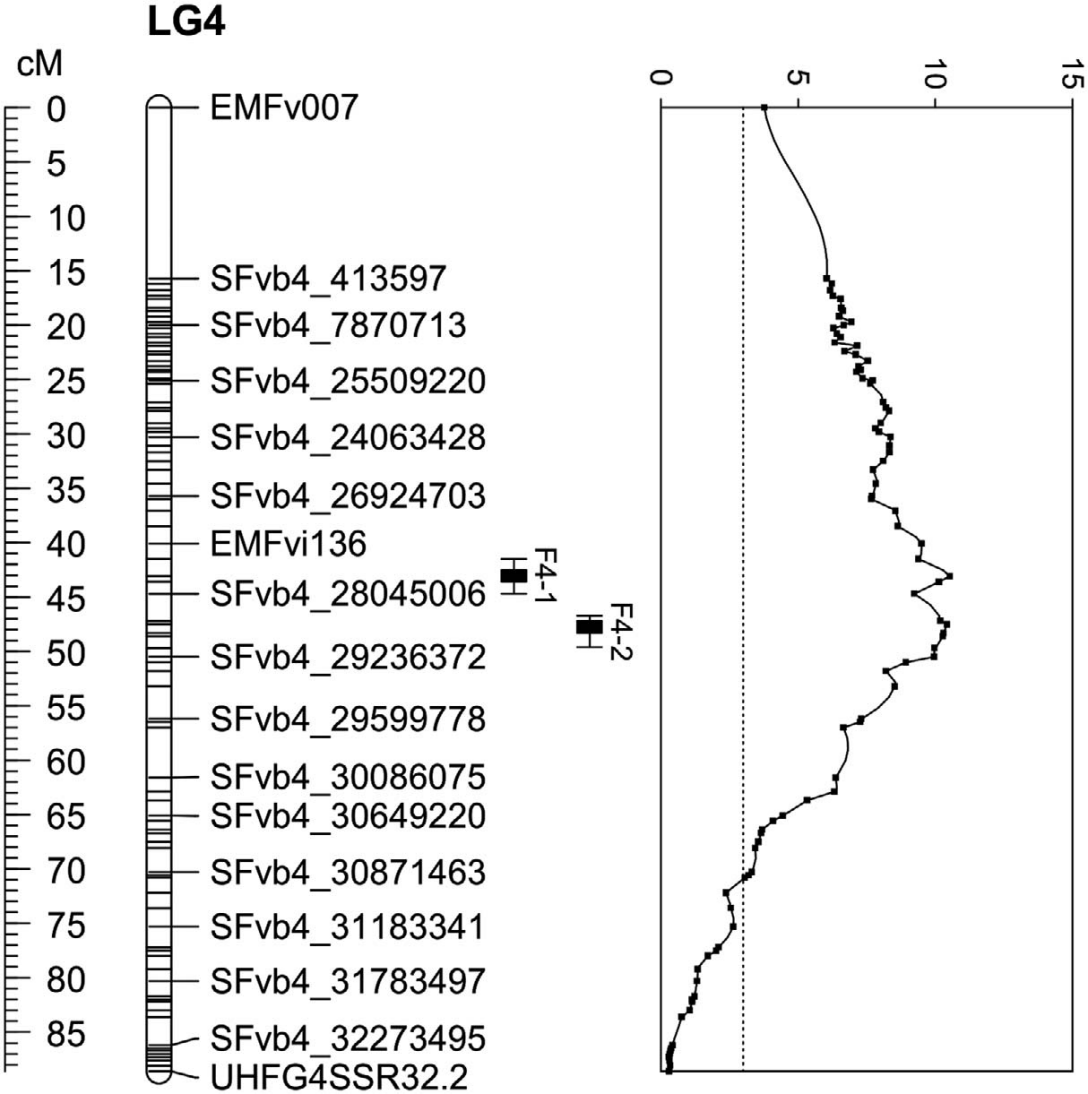
Flowering time QTL on the chromosome 4 in the field experiment. The genetic map (left) and LOD scores (right) are shown. Bars denote areas with the highest LOD scores (QTL regions). H4×FV F2 lines (*n*=186) were grown in the field and flowering time was observed in summer 2011.

The analysis of field phenotypes for flowering time revealed highly significant QTL only on LG4 (Figure 5). The peak of the LOD of this QTL was associated with marker bx083 as in the greenhouse dataset, but there was also another equally high peak ~1 Mb upstream of bx083. This QTL explained almost 25% of the phenotypic variance. Another minor QTL just above the genome-wide significance threshold of 3 was detected on LG2 with a peak of the LOD associated with the marker CFVCT020 at 11.94 cM. However, no QTLs were found on LG6 or LG7 in the field using phenotypic data of 186 F2 lines.

### Runnering and branch crown evaluation

**Figure 6:**
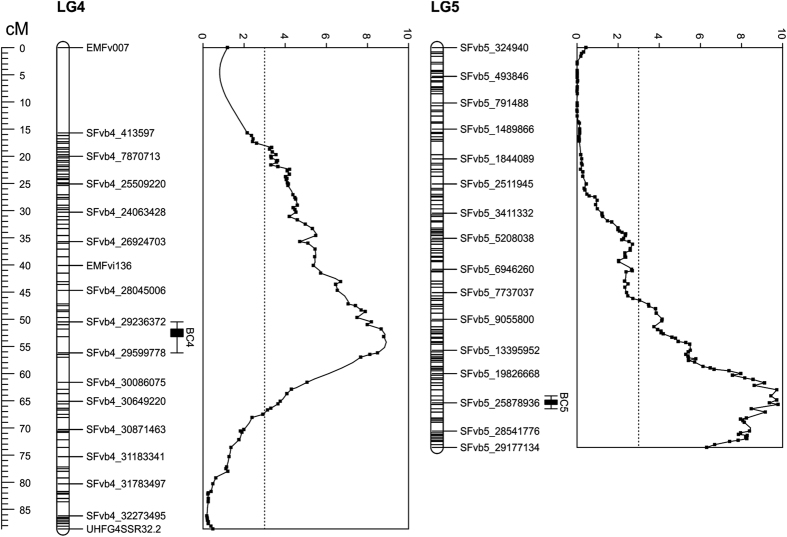
QTLs for the number of branch crowns in the greenhouse experiment. Two QTLs for the number of branch crowns (BC4 and BC5) were detected in chromosomes 4 and 5. The genetic map (left) and LOD scores (right) are shown for each chromosome. Bars denote areas with the highest LOD scores (QTL regions). Field-grown plants were taken into the greenhouse in the middle of December 2011 for growth observations. Four clones of each of 186 F2 plants were phenotyped and mean values were used for the QTL analysis.

QTL were revealed on LG4 and LG5 associated with branch crown formation and runnering. The peak of the LOD of the significant QTL for the number of branch crowns was associated with the marker SFvb4_29399865 at 53.184 cM on LG4 and with the marker SFvb_25913832 at 65.718 cM on LG5 (Figures 6a and b), and QTLs for the number of runners were located in the same regions (Supplementary Figure 5). Both QTLs explained over 20% of the variance in the number of branch crowns, but their effect on runnering was less pronounced (Table 2). Increased numbers of runners and branch crowns were associated with ‘FV’ alleles on both LG4 and LG5.

### Candidate genes for QTLs

To identify candidate genes around the QTLs, BLAST searches were carried out on the genomic regions between the flanking markers. In most cases, no obvious candidate genes were detected inside the narrow QTL peaks, but promising candidates were found very close to the highest LOD values. *FvFT2* (mrna04680.1-v1.0-hybrid) that was also identified in another QTL mapping study,^69^ was found within the ~400 kb QTL region in the end of the LG4. However, no candidate genes were searched for the other QTL on the LG4 because the physical position of the QTL was uncertain. On LG7, a homolog of *EARLY FLOWERING 6* (*ELF6*, gene23255-v1.0-hybrid)^70^ was located about 600 kb upstream of the first QTL peak, and a gene encoding a homolog of floral repressor TFL1 (*FvCENTRORADIALIS1*, *FvCEN1*; gene13304-v1.0-hybrid)^32^ was found ~650 kb upstream of the second QTL peak.

Candidate genes were also identified within the QTL intervals controlling branch crown and runner formation. On LG4, a TCP transcription factor (FvTCP7, gene04759-v1.0-hybrid)^71^ was found 150 kb upstream of the QTL peak. Furthermore, genes encoding two closely related MADS transcription factors that are homologous to DORMANCY ASSOCIATED MADS BOX (DAM, mrna12119.1-v1.0-hybrid, mrna12120.1-v1.0-hybrid),^2,72,73^ were found inside the QTL region on the LG5. No non-synonymous variation was found in the coding sequence of any candidate gene between the parental genome sequences (data not shown).

## Discussion

Extension of the strawberry production season in the open field is of significant economic importance, and two different breeding strategies have been implemented to maximize the length of the season. Several breeding programs are focusing on EB cultivars that can produce berries for several months during a single season,^6,8,42^ whereas the breeding of early and late ripening cultivars is an alternative approach to extend the ripening season.^7^ Previous QTL mapping studies in strawberry have focused on the perpetual flowering habit,^5,6,8,42^ and no QTLs have been reported for earliness. Here, we report the identification of additive QTLs on three LGs that explain over 50% of the observed 18-day variance in flowering time in woodland strawberry mapping population previously reported by Koskela *et al.*^32^ Moreover, we have found two QTLs that affect branch crown formation, the trait that has a direct effect on strawberry yield potential.^45,47,48^

### Additive QTLs on three linkage groups control flowering time in woodland strawberry

QTL mapping using replicated greenhouse flowering time data in our H4×FV F2 population revealed one new QTL on the LG4, two on the LG7, and two additional QTLs that co-localized with previously mapped QTLs on the LG4 and LG6. Four of these QTLs were also detected in a replicated growth chamber experiment using 32 F2 selected lines that exhibited extreme flowering time phenotypes in a greenhouse experiment, indicating that these QTLs are robust at least in controlled climate. In the non-replicated field experiment, the analysis using GBS data on 186 lines revealed major QTL only on LG4. However, QTL mapping on a larger population of 335 lines using SSR markers showed LOD values close to 4.0 in both LG6 and LG7 (data not shown), but a replicated field experiment is needed to confirm these QTLs in the field.

On the LG6, the QTL mapped on the same region than a previously characterized *FvTFL1* gene that encodes a repressor of flowering.^32^ In cultivated strawberry, a major QTL for perpetual flowering and runnering (*PFRU*) was previously identified on the LG4.^6,8,42^ A recent fine-mapping study placed this QTL close to marker bx083,^69^ and the same marker was located in the QTL peak also in our greenhouse and field experiments. Therefore, our data suggest a new role for *PFRU* in the control of earliness, in addition to its role in the flowering period, at least in woodland strawberry. The effect of *PFRU* on flowering time seems to be robust in different environments. However, the other QTL, that is located upstream of *PFRU* on LG4, was only detected in controlled climate experiments indicating that it may function only in specific environments.

We found other QTLs on LG7 where no flowering related QTLs have been previously detected in strawberries. These QTLs showed high LOD values in the greenhouse experiment with the F2 population of 186 lines (GBS data), but not in the field indicating that they were also influenced by the environment. One of these QTLs matched also with the QTL region detected in our growth chamber experiment (Table 1). This QTL was associated with the marker BFaCT044 at 22 166 857 bp on LG7 in a set of 32 early and late flowering lines in both growth chamber and greenhouse, and about 400 kb upstream (the most significant marker SFvb7_21710529) in the GBS dataset of 186 lines.

The analysis of allele combinations on QTL positions demonstrated their additive effects on flowering time (Figure 4; Table 2). Clearly, ‘H4’ specific markers on LG4 and LG7 QTL regions, in both heterozygous and homozygous condition delayed flowering. Furthermore, homozygous *FvTFL1* alleles from ‘FV’ parent caused additional delay compared to heterozygous plants. Plants homozygous for ‘H4’ alleles of *FvTFL1* were excluded from this study because they exhibit perpetual flowering habit.^32^

### Candidate genes for flowering time QTLs

We searched for candidate flowering time genes in the genomic regions containing the markers with the highest LOD if the physical and genetic maps on the region were mostly co-linear (Figure 2). This was not the case for the newly identified QTL on the LG4, and therefore, a better genome assembly is needed to enable reliable candidate gene search on this region.

Candidate genes for *PFRU*, that co-localize with the QTL identified in our greenhouse and field studies on the LG4, has been recently reported.^69^ These genes include *FvFT2* and *FvCDF2* that are homologs of known regulators of photoperiodic flowering. In *Arabidopsis*, CDFs are floral repressors that contribute to photoperiodic flowering by down-regulating both *CO* and *FT* mRNA expression especially in the morning.^74^ Furthermore, specific alleles of potato *CDF* gene were selected during domestication allowing the cultivation of this SD crop in LD conditions in northern latitudes.^75^ Tissue specific gene expression analyses in woodland strawberry showed that *FvFT2* is mainly expressed in flowers and fruits.^32,76^ However, both FvFT2 and FvCDF2 may also have important roles in the control of photoperiodic development in strawberries and are thus good candidates for future functional studies.

*FvELF6* was identified as a candidate gene for the QTL on LG7. ELF6 was originally identified as a repressor of photoperiodic flowering in *Arabidopsis*.^70^ It is a histone demethylase that control gene expression through chromatin regulation.^77,78^ As histone methylation is a common mechanism in the control of flowering time,^79^ and a homologous gene to *ELF6* co-localizes with a QTL for spring heat requirement for flowering on almond LG2,^2^ thus, *ELF6* is an interesting candidate for future studies.

The homolog of *TFL1*/*CEN* is located in the QTL region for flowering time in peach ^72,80^ and gene functional studies showed that *TFL1* homologs encode major floral repressors in several rosaceous species.^32–35^ Interestingly, *TFL1*/*CEN* homologs were identified as candidate genes for two QTL regions in this study. A QTL on the LG6 co-localize with *FvTFL1* that encodes a major floral repressor,^32^ and closely related *FvCEN1* is located near the second QTL peak on LG7 and awaits for functional validation. Our finding that plants containing only one functional *FvTFL1* copy flowered earlier than plants homozygous for functional ‘FV’ alleles provides interesting opportunities to control flowering time, particularly in polyploid crops such as the octoploid cultivated strawberry. In fact, a recent report showed that FaTFL1 is a major floral repressor also in cultivated strawberry, and that altered regulation of *FaTFL1* mRNA expression is associated with different flowering responses in this species.^43^ To enable more efficient breeding of flowering time, further studies are needed to uncover whether the variation in *FaTFL1* expression between cultivars is caused by allelic variation of *FaTFL1* itself or by upstream regulators. Among these regulators, the expression of *FT1* and *SOC1* homologs correlate with *TFL1* mRNA levels only in specific conditions in woodland and cultivated strawberries suggesting that *TFL1* alleles or its other regulators should be major targets of future investigations.^39,43^ Whether some of the candidate genes identified in this study control flowering time through *FvTFL1* is an interesting open question.

### Identification of QTLs controlling crown branching and runner formation

Although we mainly focused on flowering time, we also identified two QTLs on the LG4 and LG5 controlling the number of branch crowns and runners in woodland strawberry. These traits showed a strong positive correlation because the production of branch crowns increases the number of axillary buds that are able to form runners under LD conditions. Therefore, these QTL primarily control the branching of the leaf rosette in woodland strawberry. On the LG4, the QTL co-localized with previously mapped *PFRU* locus.^6,69^ However, the peak of the LOD was located about 700 kb downstream of the flowering time QTL mapped in this study. This indicates that two closely located QTLs may control flowering and axillary bud differentiation to runners and branch crowns in our population, but additional studies are needed to confirm this hypothesis.

We identified a gene encoding TCP transcription factor close to the most significant marker on the LG4. This TCP, recently named as FvTCP7,^71^ shows the highest similarity with *Arabidopsis* TCP14 and TCP15, which control cell proliferation and internode elongation.^81^ As runners are long shoots with elongated internodes this is a promising candidate gene for the strawberry axillary bud differentiation. Initial characterization of *FvTCP7* indicated that it is highly expressed in vegetative tissues including runners, but also in flower buds, and the protein is localized to nucleus.^71^ However, the function of FvTCP7 in strawberry is unknown. Further studies are needed to elucidate what is the relationship between the branching QTL detected here and *PFRU* that controls runner formation, in addition to perpetual flowering, in cultivated strawberry.^6,8^

Also the newly identified QTL region on the LG5 contained interesting candidate genes for future studies including two genes homologous to *DAM* and *SHORT VEGETATIVE PHASE* (*SVP*) that encode major regulators of dormancy in Rosaceae and floral repressor in *Arabidopsis*, respectively.^2,72,73,82^ Although strawberries do not have true dormancy, their growth vigor is strongly reduced under SDs in autumn including the cessation of runner formation, and a long period of chilling is needed to resume normal vegetative development.^83^ Functional studies would reveal whether identified FvDAM transcription factors control dormancy and what is their role in the control of axillary bud differentiation. Furthermore, the possible interaction of the branching/runnering QTLs identified in this study with the FvSOC1 and gibberellin pathway, that were previously shown to control runnering in woodland strawberry,^37,49^ should be elucidated.

## Conclusions

Here, we have reported the mapping of three new flowering time QTLs in woodland strawberry, which co-localize with homologs of known flowering time regulators that have not been functionally characterized in this species. In addition, we have shown that *PFRU*, another QTL that was previously reported to control the duration of flowering and the number of runners in cultivated strawberry,^6,69^ affects flowering time, crown branching and runner formation in woodland strawberry. Although we were able to detect four out of five flowering time QTLs in two independent experiments in controlled climate, only one of these showed a LOD value above 3 in the non-replicated field experiment. Therefore, further replicated experiments are needed to reveal the role of these QTLs in different environments. On the basis of the evidence shown here, identified QTLs control flowering time additively along with a previously identified floral repressor FvTFL1 that has quantitative effect on flowering time. As *TFL1* homologs likely integrate both light and temperature signals to control flowering time in woodland and cultivated strawberries,^39,43^ we suggest that candidate genes on the LG4 and LG7 QTL regions may control flowering time through *TFL1*. However, further studies are needed to clarify causal genes and to reveal their roles in the control of flowering time and vegetative traits. As cultivated strawberry is an octoploid species with at least two subgenome donors,^27,84^ we hypothesize that it may contain significant genetic variation in these regulators that may enable fine tuning of flowering time and shoot architecture in different environments through marker assisted selection or genomic selection strategies that have recently been developed in this species.^85^

## Figures and Tables

**Table 1:** Flowering time QTLs mapped in 32 F2 lines with extreme phenotypes

*Marker*	*Linkage group*	*Position (cM)*	*LOD value, growth chamber*	*LOD value, greenhouse*
UHFG4M19^a^	LG4	18.91	3.8	6.42
bx083	LG4	37.70	4.1	7.42
TFL1	LG6	17.84	5.01	13.59
BFaCT44	LG7	40.59	3.57	5.59

a Markers at 15.91–20.17 cM showed the same LOD value.

The most significant markers for each QTL, their positions and LOD values in growth chamber and greenhouse experiments are shown.

**Table 2:** QTLs mapped in the greenhouse experiment

*QTL/ phenotype*	*Marker*	*Position (bp)*	*Position (cM)*	*LOD value*	*Explained variance (%)*	*Dominance*	*Additive*
FT	SFvb4_7870713	Fvb4: 7870713	20.012	7.1	16.1	−0.137	1.807
FT	Bx083/ SFvb4_28667943	Fvb4: 28667943	47.485	6.58	15	0.427	1.784
FT	TFL1	Fvb6: 10371025	40.306	7.59	17	−4.380	1.623
FT	SFvb7_16204472	Fvb7: 16204472	24.895	5.45	12.6	0.232	1.775
FT	SFvb7_21710529	Fvb7: 21710529	14.215	5.16	12	−0.212	1.617
BC	SFvb4_29399865	Fvb4: 29399865	53.184	8.8	20.5	−0.053	−0.552
BC	SFvb5_25913832	Fvb5: 25913832	65.718	9.78	22.5	0.151	−0.582
RU	SFvb4_29399865	Fvb4: 29399865	53.184	6.56	15	0.536	−1.382
RU	SFvb5_25814268	Fvb5: 25814268	64.908	6.13	14.1	0.899	−1.271

Abbreviations: BC, branch crowns; FT, flowering time; RU, runners

The most significant markers for each QTL, their positions and different genetic parameters are shown. Negative or positive value for dominance indicate that the allele of ‘H4’ or ‘FV’ parent has larger effect on the phenotype, respectively.

## References

[bib1] Andrés F, Coupland G. The genetic basis of flowering responses to seasonal cues. Nat Rev Genet 2012; 13: 627–639. 22898651 10.1038/nrg3291

[bib2] Sánchez-Pérez R, Dicenta F, Martinez-Gomez P. Inheritance of chilling and heat requirements for flowering in almond and QTL analysis. Tree Genet Genomes 2012; 8: 379–389.

[bib3] Hibrand-Saint Oyant L, Crespel L, Rajapakse S, Zhang L, Foucher F. Genetic linkage maps of rose constructed with new microsatellite markers and locating QTL controlling flowering traits. Tree Genet Genomes 2007; 4: 11–23.

[bib4] Zhebentyayeva TN, Fan S, Chandra A et al. Dissection of chilling requirement and bloom date QTLs in peach using a whole genome sequencing of sibling trees from an F2 mapping population. Tree Genet Genomes 2014; 10: 35–51.

[bib5] Weebadde CK, Wang D, Finn C et al. Using a linkage mapping approach to identify QTL for day-neutrality in the octoploid strawberry. Plant Breed 2008; 127: 94–101.

[bib6] Gaston A, Perrotte J, Lerceteau-Köhler E et al. PFRU, a single dominant locus regulates the balance between sexual and asexual plant reproduction in cultivated strawberry. J Exp Bot 2013; 64: 1837–1848. 23554259 10.1093/jxb/ert047

[bib7] Bestfleisch M, Möhring J, Hanke MV, Peil A, Flachowsky H. A diallel crossing approach aimed on selection for ripening time and yield in breeding of new strawberry (Fragaria×ananassa Duch.) cultivars. Plant Breed 2014; 133: 115–120.

[bib8] Castro P, Bushakra JM, Stewart P et al. Genetic mapping of day-neutrality in cultivated strawberry. Mol Breed 2015; 35: 1–16.

[bib9] Opstad N, Sønsteby A, Myrheim U, Heide OM. Seasonal timing of floral initiation in strawberry: effects of cultivar and geographic location. Sci Hortic 2011; 129: 127–134.

[bib10] Heide OM. Photoperiod and temperature interactions in growth and flowering of strawberry. Physiol Plant 1977; 40: 21–26.

[bib11] Durner EF. Photoperiod and temperature effects on flower and runner development in day-neutral, Junebearing, and everbearing strawberries. J Amer Soc Hort Sci 1984; 109: 396–400.

[bib12] Manakasem Y, Goodwin PB. Responses of dayneutral and Junebearing strawberries to temperature and daylength. J Hortic Sci Biotechnol 2001; 76: 629–635.

[bib13] Heide OM, Sønsteby A. Interactions of temperature and photoperiod in the control of flowering of latitudinal and altitudinal populations of wild strawberry (*Fragaria vesca*). Physiol Plant 2007; 130: 280–289.

[bib14] Bradford E, Hancock JF, Warner RM. Interactions of temperature and photoperiod determine expression of repeat flowering in strawberry. J Am Soc Hortic Sci 2010; 135: 102–107.

[bib15] Sønsteby A, Heide OM. Dormancy relations and flowering of the strawberry cultivars Korona and Elsanta as influenced by photoperiod and temperature. Sci Hortic 2006; 110: 57–67.

[bib16] Nishiyama M, Kanahama K. Effects of temperature and photoperiod on flower bud initiation of day-neutral and everbearing strawberries. Acta Hortic 2002; 567: 253–255.

[bib17] Sønsteby A, Heide O. Long-day control of flowering in everbearing strawberries. J Hortic Sci Biotechnol 2007; 82: 875–884.

[bib18] Brown T, Wareing PF. the genetical control of the everbearing habit and three other characters in varieties of *Fragaria vesca*. Euphytica 1965; 14: 97–112.

[bib19] Sargent DJ, Kuchta P, Girona EL et al. Simple sequence repeat marker development and mapping targeted to previously unmapped regions of the strawberry genome sequence. Plant Genome J 2011; 4: 165–177.

[bib20] Sargent DJ, Davis TM, Tobutt KR, Wilkinson MJ, Battey NH, Simpson DW. A genetic linkage map of microsatellite, gene-specific and morphological markers in diploid Fragaria. Theor Appl Genet 2004; 109: 1385–1391. 15290052 10.1007/s00122-004-1767-9

[bib21] Sargent DJ, Clarke J, Simpson DW et al. An enhanced microsatellite map of diploid Fragaria. Theor Appl Genet 2006; 112: 1349–1359. 16505996 10.1007/s00122-006-0237-y

[bib22] Ashley MV, Wilk JA, Styan SMN et al. High variability and disomic segregation of microsatellites in the octoploid *Fragaria virginiana* Mill. (Rosaceae). Theor Appl Genet 2003; 107: 1201–1207. 12908097 10.1007/s00122-003-1370-5

[bib23] Tabone T, Mather DE, Hayden MJ. Temperature switch PCR (TSP): robust assay design for reliable amplification and genotyping of SNPs. BMC Genomics 2009; 10: 580. 19958555 10.1186/1471-2164-10-580 PMC2795770

[bib24] Schuelke M. An economic method for the fluorescent labeling of PCR fragments. Nat Biotechnol 2000; 18: 233–234. 10657137 10.1038/72708

[bib25] Urrutia M, Bonet J, Arús P, Monfort A. A near-isogenic line (NIL) collection in diploid strawberry and its use in the genetic analysis of morphologic, phenotypic and nutritional characters. Theor Appl Genet 2015; 128: 1261–1275. 25841354 10.1007/s00122-015-2503-3

[bib26] Shulaev V, Sargent DJ, Crowhurst RN et al. The genome of woodland strawberry (*Fragaria vesca*). Nat Genet 2011; 43: 109–116. 21186353 10.1038/ng.740 PMC3326587

[bib27] Tennessen Ja, Govindarajulu R, Ashman T-L, Liston A. Evolutionary origins and dynamics of octoploid strawberry subgenomes revealed by dense targeted capture linkage maps. Genome Biol Evol 2014; 6: 3295–3313. 25477420 10.1093/gbe/evu261 PMC4986458

[bib28] Bassil NV, Davis TM, Zhang H et al. Development and preliminary evaluation of a 90 K Axiom SNP array for the allo-octoploid cultivated strawberry Fragaria×ananassa. BMC Genomics 2015; 16: 155. 25886969 10.1186/s12864-015-1310-1 PMC4374422

[bib29] Mahoney LL, Sargent DJ, Abebe-Akele F et al. A high-density linkage map of the ancestral diploid strawberry, constructed with single nucleotide polymorphism markers from the IStraw90 Array and genotyping by sequencing. Plant Genome 2016; 9: 1–14. 10.3835/plantgenome2015.08.0071 27898812

[bib30] Deng C, Davis TM. Molecular identification of the yellow fruit color (c) locus in diploid strawberry: a candidate gene approach. Theor Appl Genet 2001; 103: 316–322.

[bib31] Albani MC, Battey NH, Wilkinson MJ. The development of ISSR-derived SCAR markers around the SEASONAL FLOWERING LOCUS (SFL) in *Fragaria vesca*. Theor Appl Genet 2004; 109: 571–579. 15292991 10.1007/s00122-004-1654-4

[bib32] Koskela EA, Mouhu K, Albani MC et al. Mutation in TERMINAL FLOWER1 reverses the photoperiodic requirement for flowering in the wild strawberry *Fragaria vesca*. Plant Physiol 2012; 159: 1043–1054. 22566495 10.1104/pp.112.196659 PMC3387692

[bib33] Iwata H, Gaston A, Remay A et al. The TFL1 homologue KSN is a regulator of continuous flowering in rose and strawberry. Plant J 2012; 69: 116–125. 21895811 10.1111/j.1365-313X.2011.04776.x

[bib34] Flachowsky H, Szankowski I, Waidmann S, Peil A, Tränkner C, Hanke MV. The MdTFL1 gene of apple (Malus×domestica Borkh.) reduces vegetative growth and generation time. Tree Physiol 2012; 32: 1288–1301. 23022687 10.1093/treephys/tps080

[bib35] Freiman A, Shlizerman L, Golobovitch S et al. Development of a transgenic early flowering pear (*Pyrus communis* L.) genotype by RNAi silencing of PcTFL1-1 and PcTFL1-2. Planta 2012; 235: 1239–1251. 22203321 10.1007/s00425-011-1571-0

[bib36] Kurokura T, Mimida N, Battey NH, Hytönen T. The regulation of seasonal flowering in the Rosaceae. J Exp Bot 2013; 64: 4131–4141. 23929655 10.1093/jxb/ert233

[bib37] Mouhu K, Kurokura T, Koskela EA et al. The *Fragaria vesca* Homolog of SUPPRESSOR OF OVEREXPRESSION OF CONSTANS1 represses flowering and promotes vegetative growth. Plant Cell 2013; 25: 3296–3310. 24038650 10.1105/tpc.113.115055 PMC3809533

[bib38] Rantanen M, Kurokura T, Mouhu K et al. Light quality regulates flowering in FvFT1/FvTFL1 dependent manner in the woodland strawberry *Fragaria vesca*. Front Plant Sci 2014; 5: 271. 24966865 10.3389/fpls.2014.00271 PMC4052200

[bib39] Rantanen M, Kurokura T, Jiang P, Mouhu K, Hytönen T. Strawberry homologue of TERMINAL FLOWER1 integrates photoperiod and temperature signals to inhibit flowering. Plant J 2015; 82: 163–173. 25720985 10.1111/tpj.12809

[bib40] Ahmadi H, Bringhurst RS, Voth V. Modes of inheritance of photoperiodism in Fragaria. J Amer Soc Hort Sci 1990; 115: 146–152.

[bib41] Sugimoto T, Tamaki K, Matsumoto J, Yamamoto Y, Shiwaku K, Watanabe K. Detection of RAPD markers linked to the everbearing gene in Japanese cultivated strawberry. Plant Breed 2005; 124: 498–501.

[bib42] Honjo M, Nunome T, Kataoka S et al. Simple sequence repeat markers linked to the everbearing flowering gene in long-day and day-neutral cultivars of the octoploid cultivated strawberry Fragaria×ananassa. Euphytica 2016; 209: 291–303.

[bib43] Koskela E, Sønsteby A, Flachowsky H et al. TERMINAL FLOWER1 is a breeding target for a novel everbearing trait and tailored flowering responses in cultivated strawberry (Fragaria×ananassa Duch.). Plant Biotechnol J 2016; 14: 1852–1861. 26940366 10.1111/pbi.12545 PMC5069601

[bib44] Nakano Y, Higuchi Y, Yoshida Y, Hisamatsu T. Environmental responses of the FT/TFL1 gene family and their involvement in flower induction in Fragaria×ananassa. J Plant Physiol 2015; 177: 60–66. 25666540 10.1016/j.jplph.2015.01.007

[bib45] Konsin M, Voipio I, Palonen P. Influence of photoperiod and duration of short-day treatment on vegetative growth and flowering of strawberry (Fragaria×ananassa duch). J Hortic Sci Biotechnol 2001; 76: 77–82.

[bib46] de Camacaro MEP, Camacaro GJ, Hadley P, Battey NH, Carew JG. Pattern of growth and development of the strawberry cultivars Elsanta, Bolero, and Everest. J Am Soc Hortic Sci 2002; 127: 901–907.

[bib47] Hytönen T, Palonen P, Mouhu K, Junttila O. Crown branching and cropping potential in strawberry (Fragaria×ananassa Duch.) can be enhanced by daylength treatments. J Hortic Sci Biotechnol 2004; 79: 466–471.

[bib48] Hytönen T, Mouhu K, Koivu I, Junttila O. Prohexadione-calcium enhances the cropping potential and yield of strawberry. Eur J Hortic Sci 2008; 73: 210–215.

[bib49] Hytönen T, Elomaa P, Moritz T, Junttila O. Gibberellin mediates daylength-controlled differentiation of vegetative meristems in strawberry (Fragaria×ananassa Duch). BMC Plant Biol 2009; 9: 18. 19210764 10.1186/1471-2229-9-18 PMC2653492

[bib50] Cipriani G, Testolin R. Isolation and characterization of microsatellite loci in Fragaria. Mol Ecol Notes 2004; 4: 366–368.

[bib51] Monfort A, Vilanova S, Davis TM, Arus P. A new set of polymorphic simple sequence repeat (SSR) markers from a wild strawberry (*Fragaria vesca*) are transferable to other diploid Fragaria species and to Fragaria x ananassa. Mol Ecol Notes 2006; 6: 197–200.

[bib52] Sargent DJ, Hadonou AM, Simpson DW. Development and characterization of polymorphic microsatellite markers from Fragaria viridis, a wild diploid strawberry. Mol Ecol Notes 2003; 3: 550–552.

[bib53] Sargent DJ, Passey T, Surbanovski N et al. A microsatellite linkage map for the cultivated strawberry (Fragaria×ananassa) suggests extensive regions of homozygosity in the genome that may have resulted from breeding and selection. Theor Appl Genet 2012; 124: 1229–1240. 22218676 10.1007/s00122-011-1782-6

[bib54] Rozen S, Skaletsky H. Primer3 on the WWW for general users and for biologist programmers. Methods Mol Biol 1999; 132: 365–386. 10.1385/1-59259-192-2:365 10547847

[bib55] Elshire RJ, Glaubitz JC, Sun Q et al. A robust, simple genotyping-by-sequencing (GBS) approach for high diversity species. PLoS ONE 2011; 6: e19379. 21573248 10.1371/journal.pone.0019379 PMC3087801

[bib56] Glaubitz JC, Casstevens TM, Lu F et al. TASSEL-GBS: a high capacity genotyping by sequencing analysis pipeline. PLoS ONE 2014; 9: e90346. 24587335 10.1371/journal.pone.0090346 PMC3938676

[bib57] Bradbury PJ, Zhang Z, Kroon DE, Casstevens TM, Ramdoss Y, Buckler ES. TASSEL: software for association mapping of complex traits in diverse samples. Bioinformatics 2007; 23: 2633–2635. 17586829 10.1093/bioinformatics/btm308

[bib58] Danecek P, Auton A, Abecasis G et al. The variant call format and VCFtools. Bioinformatics 2011; 27: 2156–2158. 21653522 10.1093/bioinformatics/btr330 PMC3137218

[bib59] Browning BL, Browning SR. Improving the accuracy and efficiency of identity-by-descent detection in population data. Genetics 2013; 194: 459–471. 23535385 10.1534/genetics.113.150029 PMC3664855

[bib60] Ward JA, Bhangoo J, Fernández-Fernández F et al. Saturated linkage map construction in Rubus idaeus using genotyping by sequencing and genome-independent imputation. BMC Genomics 2013; 14: 2. 23324311 10.1186/1471-2164-14-2 PMC3575332

[bib61] Van Ooijen JW. JoinMap 4, Software for the Calculation of Genetic Linkage Maps in Experimental Populations. Kyazma B.V.: Wageningen, Netherlands, 2006.

[bib62] Voorrips RE. MapChart: software for the graphical presentation of linkage maps and QTLs. J Hered 2002; 93: 77–78. 12011185 10.1093/jhered/93.1.77

[bib63] Van Ooijen JW. MapQTL 5, Software for the Mapping of Quantitative Trait Loci in Experimental Populations. Kyazma B.V.: Wageningen, Netherlands, 2004.

[bib64] Martin M. Cutadapt removes adapter sequences from high-throughput sequencing reads. EMBnet.journal 2011; 17: 10.

[bib65] Li H. Aligning sequence reads, clone sequences and assembly contigs with BWA-MEM. arXiv Prepr arXiv13033997; 2013.

[bib66] Li H, Handsaker B, Wysoker A et al. The sequence alignment/Map format and SAMtools. Bioinformatics 2009; 25: 2078–2079. 19505943 10.1093/bioinformatics/btp352 PMC2723002

[bib67] Li H. A statistical framework for SNP calling, mutation discovery, association mapping and population genetical parameter estimation from sequencing data. Bioinformatics 2011; 27: 2987–2993. 21903627 10.1093/bioinformatics/btr509 PMC3198575

[bib68] Conesa A, Götz S, García-Gómez JM, Terol J, Talón M, Robles M. Blast2GO: a universal tool for annotation, visualization and analysis in functional genomics research. Bioinformatics 2005; 21: 3674–3676. 16081474 10.1093/bioinformatics/bti610

[bib69] Perrotte J, Gaston A, Potier A, Petit A, Rothan C, Denoyes B. Narrowing down the single homoeologous FaPFRU locus controlling flowering in cultivated octoploid strawberry using a selective mapping strategy. Plant Biotechnol J 2016; 14: 2176–2189. 27168086 10.1111/pbi.12574 PMC5095798

[bib70] Noh B, Lee S-H, Kim H-J et al. Divergent roles of a pair of homologous jumonji/zinc-finger-class transcription factor proteins in the regulation of *Arabidopsis* flowering time. Plant Cell 2004; 16: 2601–2613. 15377760 10.1105/tpc.104.025353 PMC520958

[bib71] Wei W, Hu Y, Cui M-Y, Han Y-T, Gao K, Feng J-Y. Identification and transcript analysis of the TCP transcription factors in the diploid woodland strawberry *Fragaria vesca*. Front Plant Sci 2016; 7: 1–18. 28066489 10.3389/fpls.2016.01937 PMC5177655

[bib72] Fan S, Bielenberg DG, Zhebentyayeva TN et al. Mapping quantitative trait loci associated with chilling requirement, heat requirement and bloom date in peach (*Prunus persica*). N Phytol 2010; 185: 917–930. 10.1111/j.1469-8137.2009.03119.x 20028471

[bib73] Bielenberg DG, Wang Y, Li Z et al. Sequencing and annotation of the evergrowing locus in peach [*Prunus persica* (L.) Batsch] reveals a cluster of six MADS-box transcription factors as candidate genes for regulation of terminal bud formation. Tree Genet Genomes 2008; 4: 495–507.

[bib74] Song YH, Smith RW, To BJ, Miller AJ, Imaizumi T, Millar AJ. FKF1 conveys timing information for CONSTANS stabilisation in photoperiodic flowering. Science 2012; 336: 1045–1049. 22628657 10.1126/science.1219644 PMC3737243

[bib75] Kloosterman B, Abelenda JA, del Mar Carretero Gomez M, Oortwijn M, de Boer JM, Kowitwanich K. Naturally occurring allele diversity allows potato cultivation in northern latitudes. Nature 2013; 495: 246–250. 23467094 10.1038/nature11912

[bib76] Kang C, Darwish O, Geretz A, Shahan R, Alkharouf N, Liu Z. Genome-scale transcriptomic insights into early-stage fruit development in woodland strawberry *Fragaria vesca*. Plant Cell 2013; 25: 1960–1978. 23898027 10.1105/tpc.113.111732 PMC3723606

[bib77] Jiang D, Yang W, He Y, Amasino RM. *Arabidopsis* relatives of the human lysine-specific demethylase1 repress the expression of FWA and FLOWERING LOCUS C and thus promote the floral transition. Plant Cell 2007; 19: 2975–2987. 17921315 10.1105/tpc.107.052373 PMC2174716

[bib78] Jeong JH, Song HR, Ko JH et al. Repression of FLOWERING LOCUS T chromatin by functionally redundant histone H3 lysine 4 demethylases in *Arabidopsis*. PLoS ONE 2009; 4: e8033. 19946624 10.1371/journal.pone.0008033 PMC2777508

[bib79] Jeong HJ, Yang J, Yi J, An G. Controlling flowering time by histone methylation and acetylation in *Arabidopsis* and rice. J Plant Biol 2015; 58: 203–210.

[bib80] Romeu JF, Monforte AJ, Sánchez G, Granell A, García-Brunton J, Badenes ML et al. Quantitative trait loci affecting reproductive phenology in peach. BMC Plant Biol 2014; 14: 52. 24559033 10.1186/1471-2229-14-52 PMC3941940

[bib81] Kieffer M, Master V, Waites R, Davies B. TCP14 and TCP15 affect internode length and leaf shape in *Arabidopsis*. Plant J 2011; 68: 147–158. 21668538 10.1111/j.1365-313X.2011.04674.x PMC3229714

[bib82] Hartmann U, Höhmann S, Nettesheim K, Wisman E, Saedler H, Huijser P. Molecular cloning of SVP: a negative regulator of the floral transition in *Arabidopsis*. Plant J 2000; 21: 351–360. 10758486 10.1046/j.1365-313x.2000.00682.x

[bib83] Heide OM, Stavang JA, Sønsteby A. Physiology and genetics of flowering in cultivated and wild strawberries—a review. J Hortic Sci Biotechnol 2013; 88: 1–18.

[bib84] Sargent DJ, Yang Y, Šurbanovski N et al. HaploSNP affinities and linkage map positions illuminate subgenome composition in the octoploid, cultivated strawberry (Fragaria×ananassa). Plant Sci 2016; 242: 140–150. 26566832 10.1016/j.plantsci.2015.07.004

[bib85] Gezan SA, Osorio LF, Verma S, Whitaker VM. An experimental validation of genomic selection in octoploid strawberry. Hortic Res 2017; 4: 16070. 28090334 10.1038/hortres.2016.70 PMC5225750

