# Supplementary material for: Additive QTLs on three chromosomes control flowering time in woodland strawberry (Fragaria vesca L.)
Source: Hortic Res. 2017 May 24;4:17020–. doi: 10.1038/hortres.2017.20 (PMC5442962; doi:10.1038/hortres.2017.20)
Supplement: Supplementary Figure Legends [file hortres201720-s1.pdf]

Additive QTLs on three chromosomes control flowering time in woodland strawberry (*Fragaria vesca* L.)

Samia Samad, Takeshi Kurokura, Elli Koskela, Tuomas Toivainen, Vipul Patel, Katriina Mouhu, Daniel James Sargent, Timo Hytönen

SUPPLEMENTAL FIGURES

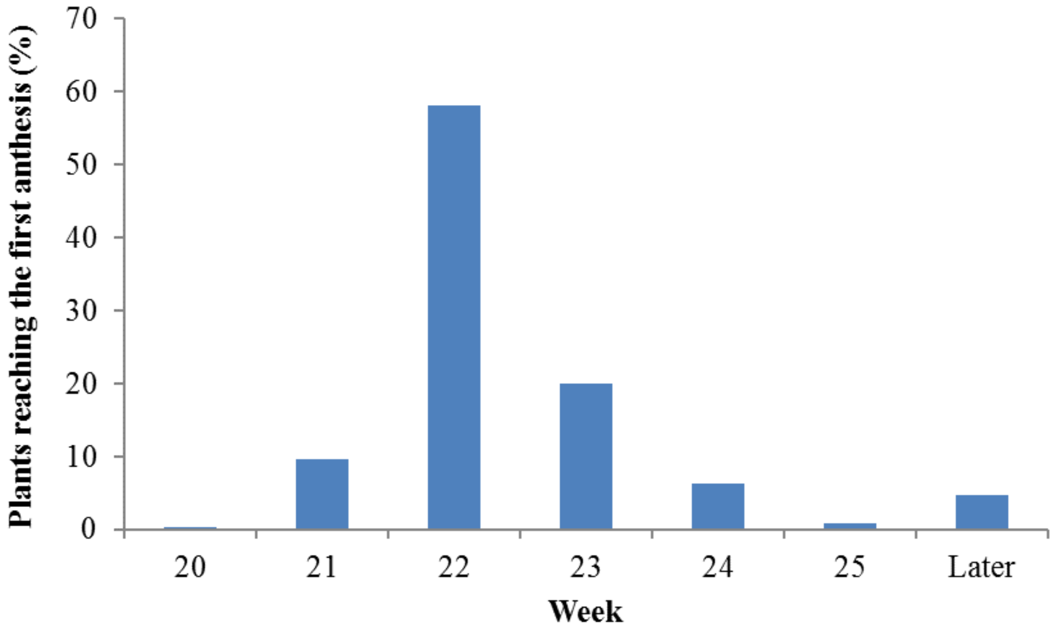

Supplemental Figure 1. Segregation of flowering time in H4×FV mapping population. The presence of open flowers were observed weekly on 735 F2 lines in the field in Helsinki in summer 2011, and the percentage of plants with the first open flower were recorded.

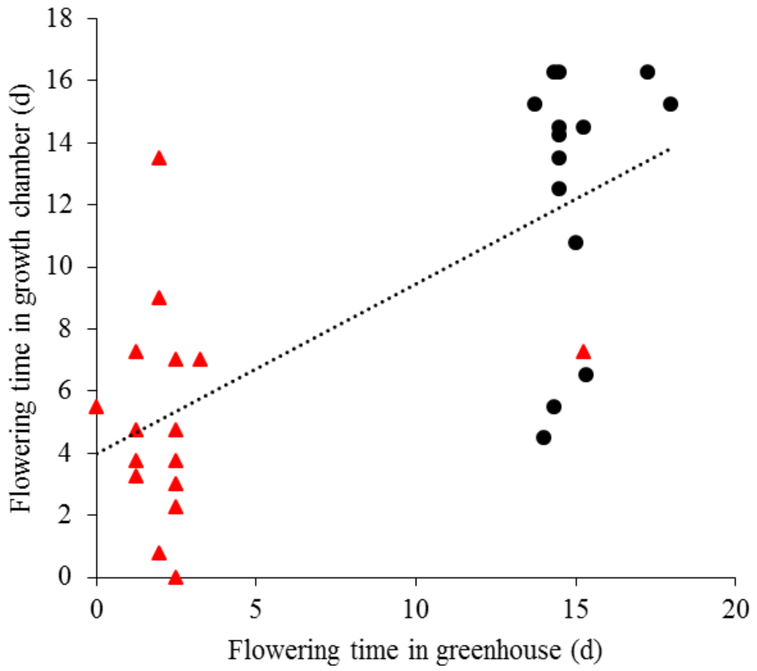

Supplemental Figure 2. Flowering time of 32 F2 lines in the greenhouse and growth chamber experiments. The 16 earliest and the 16 latest F2 lines were selected based on greenhouse phenotypic data, and the plants were subjected to short days and cool temperature for six weeks, followed by flowering observations in a greenhouse. Plants containing one or two functional FvTFL1 alleles are shown with red triangles and black circles, respectively. Both greenhouse and growth chamber values are means of four replicates.

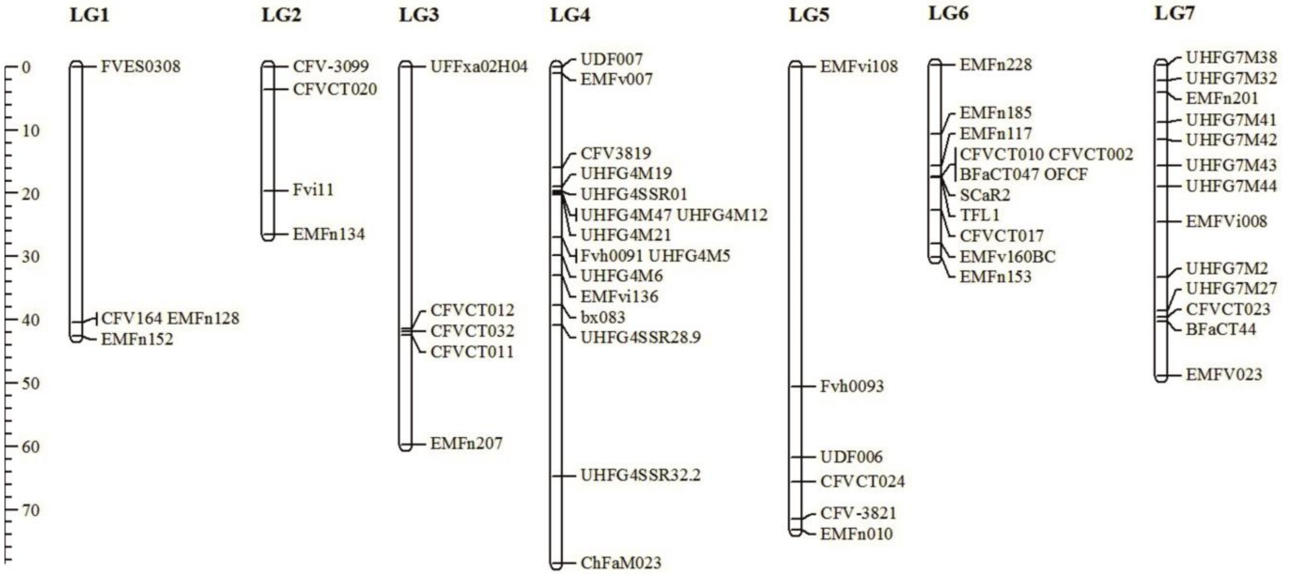

Supplemental Figure 3. Linkage map of 335 F2 progeny of the H4×FV cross comprising of 60 molecular markers. The numbers at the top denote the chromosome number and the genetic distances are in centiMorgan (scale in the left).

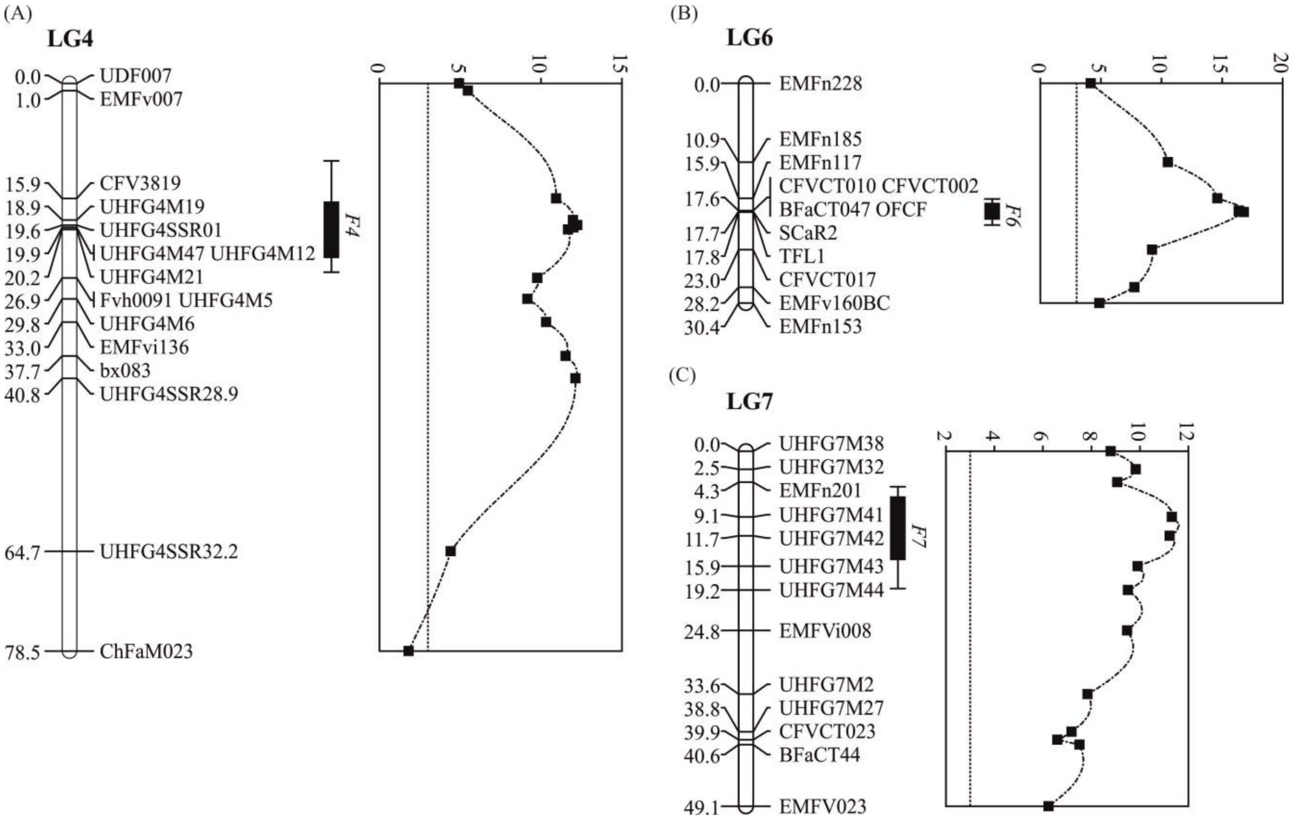

Supplemental Figure 4. Flowering time QTLs in a greenhouse experiment. Three QTLs for flowering time (F4, F6 and F7) were detected on chromosomes 4 (A), 6 (B), and 7 (C). The genetic map (left) and LOD scores (right) are shown for each chromosome. The bar denotes the area with the highest LOD score (QTL region). Field-grown plants were moved to the greenhouse in the middle of December 2011 for flowering time observations. Four clones of each of 335 F2 plants were phenotyped and mean values were used for the QTL analysis.

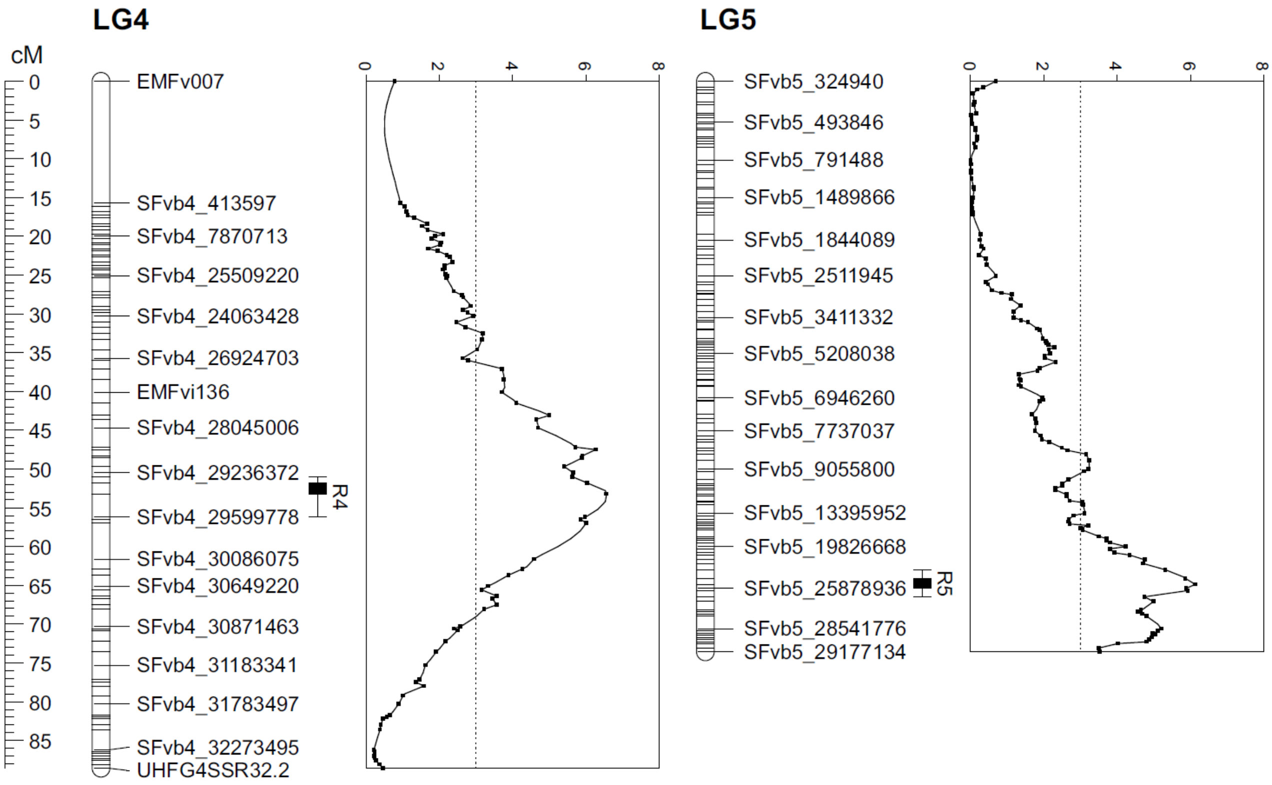

Supplemental Figure 5. QTLs for the number of runners in the greenhouse experiment. Two QTLs for the number of runners (R4 and R5) were detected in chromosomes 4 and 5. The genetic map (left) and LOD scores (right) are shown for each chromosome. Bars denote areas with the highest LOD scores (QTL regions). Field-grown plants were taken into the greenhouse in the middle of December 2011 for growth observations. Four clones of each of 188 F2 plants were phenotyped and mean values were used for the QTL analysis.
