# Supplementary figures and images for: Additive QTLs on three chromosomes control flowering time in woodland strawberry (Fragaria vesca L.)
Source: Hortic Res. 2017 May 24;4:17020–. doi: 10.1038/hortres.2017.20 (PMC5442962; doi:10.1038/hortres.2017.20)

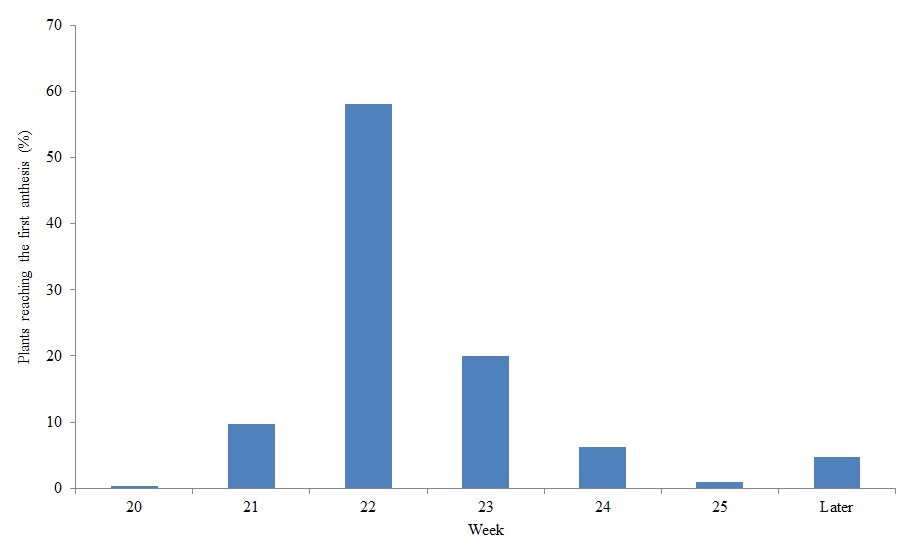

Supplement: Supplementary Figure 1 [file hortres201720-s2.jpg]

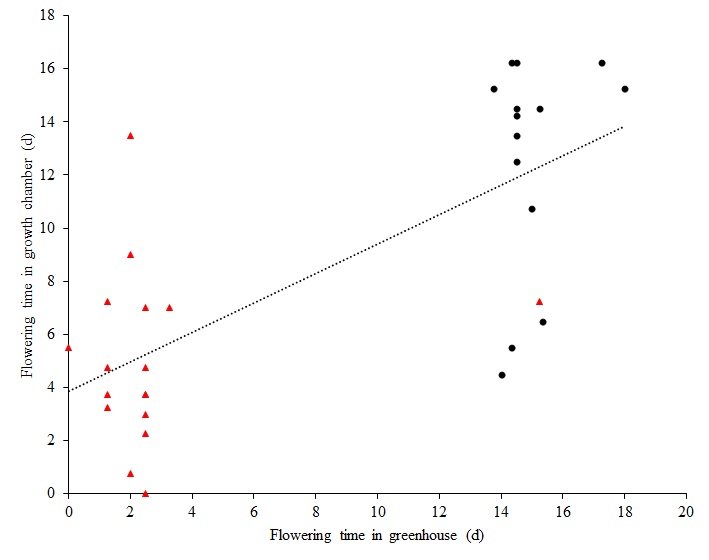

Supplement: Supplementary Figure 2 [file hortres201720-s3.jpg]

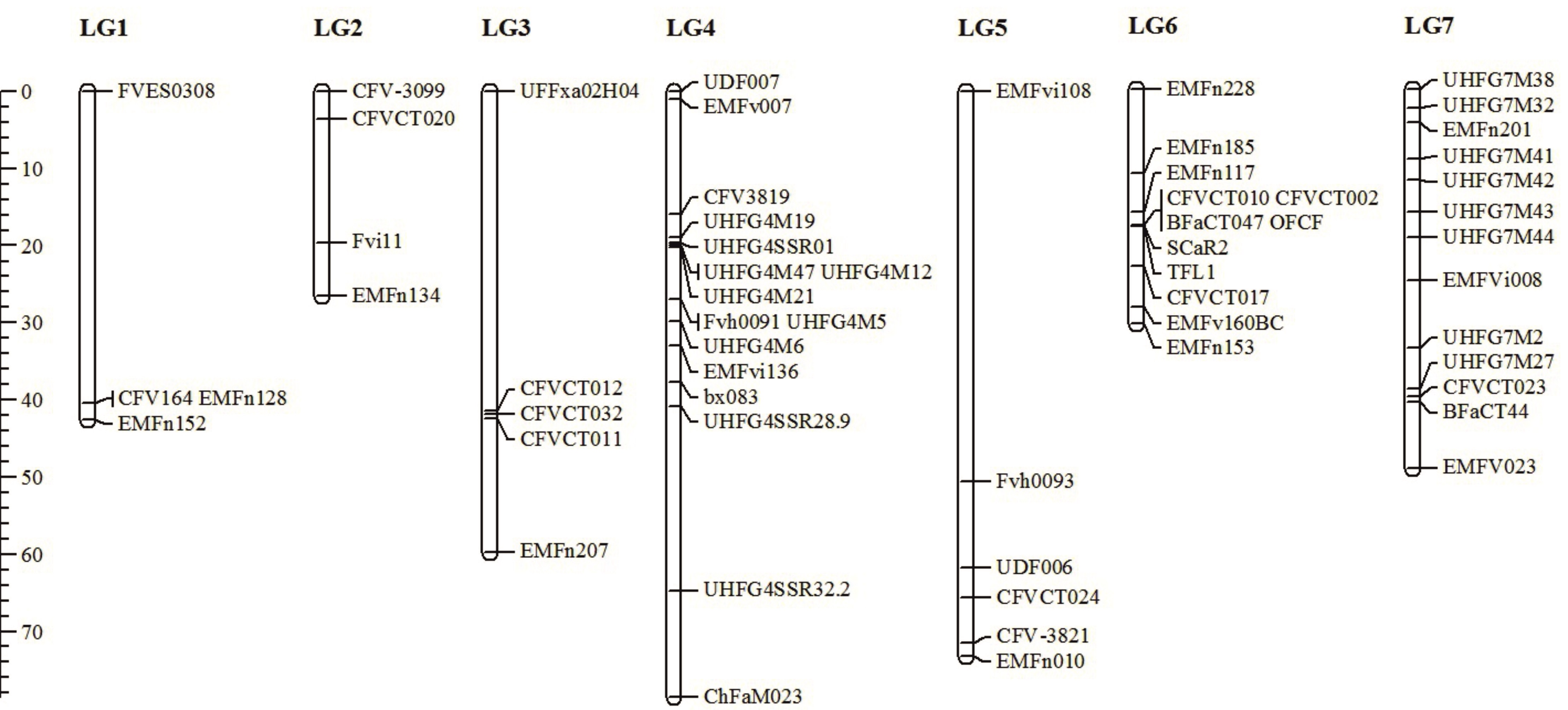

Supplement: Supplementary Figure 3 [file hortres201720-s4.jpg]

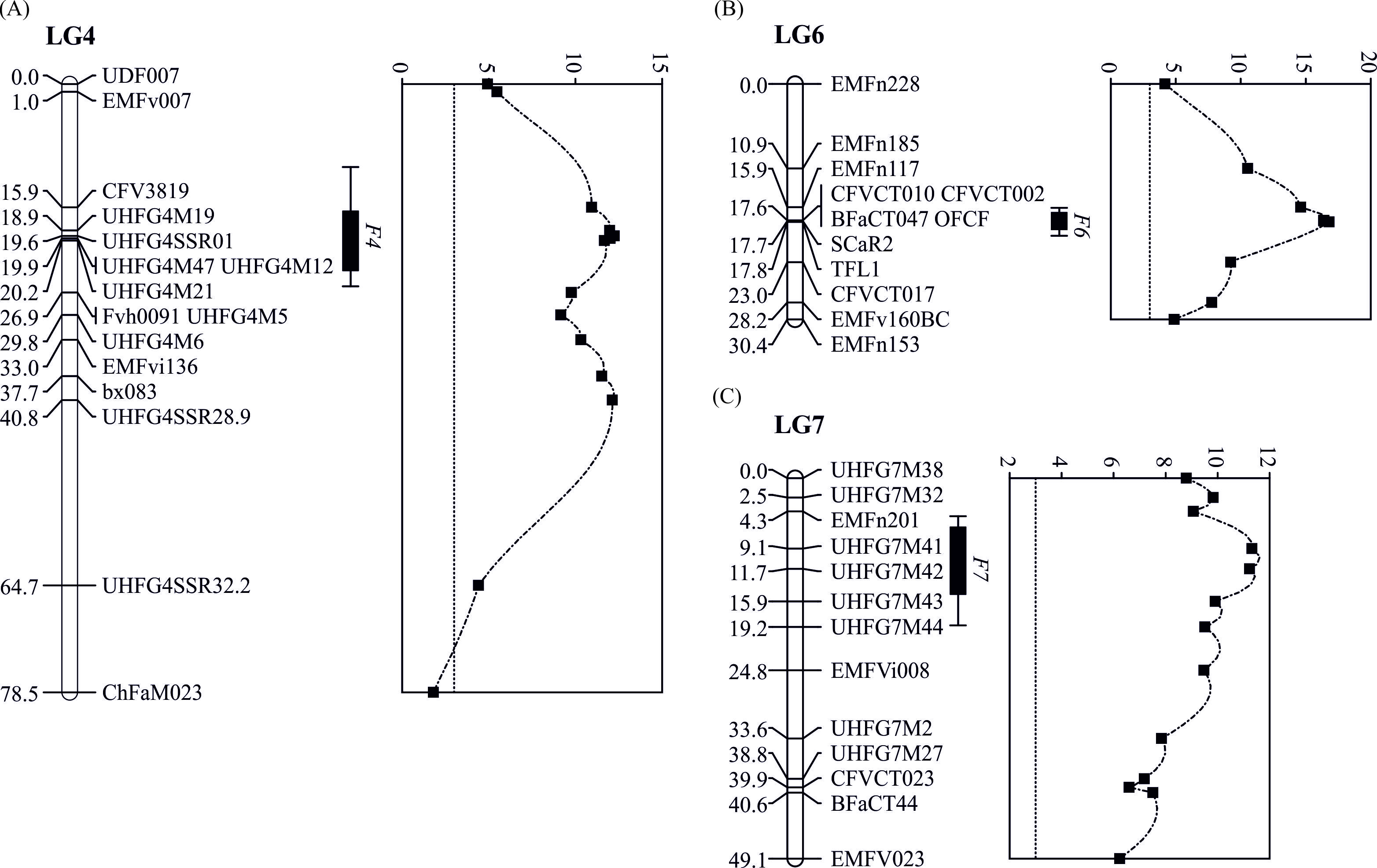

Supplement: Supplementary Figure 4 [file hortres201720-s5.jpg]

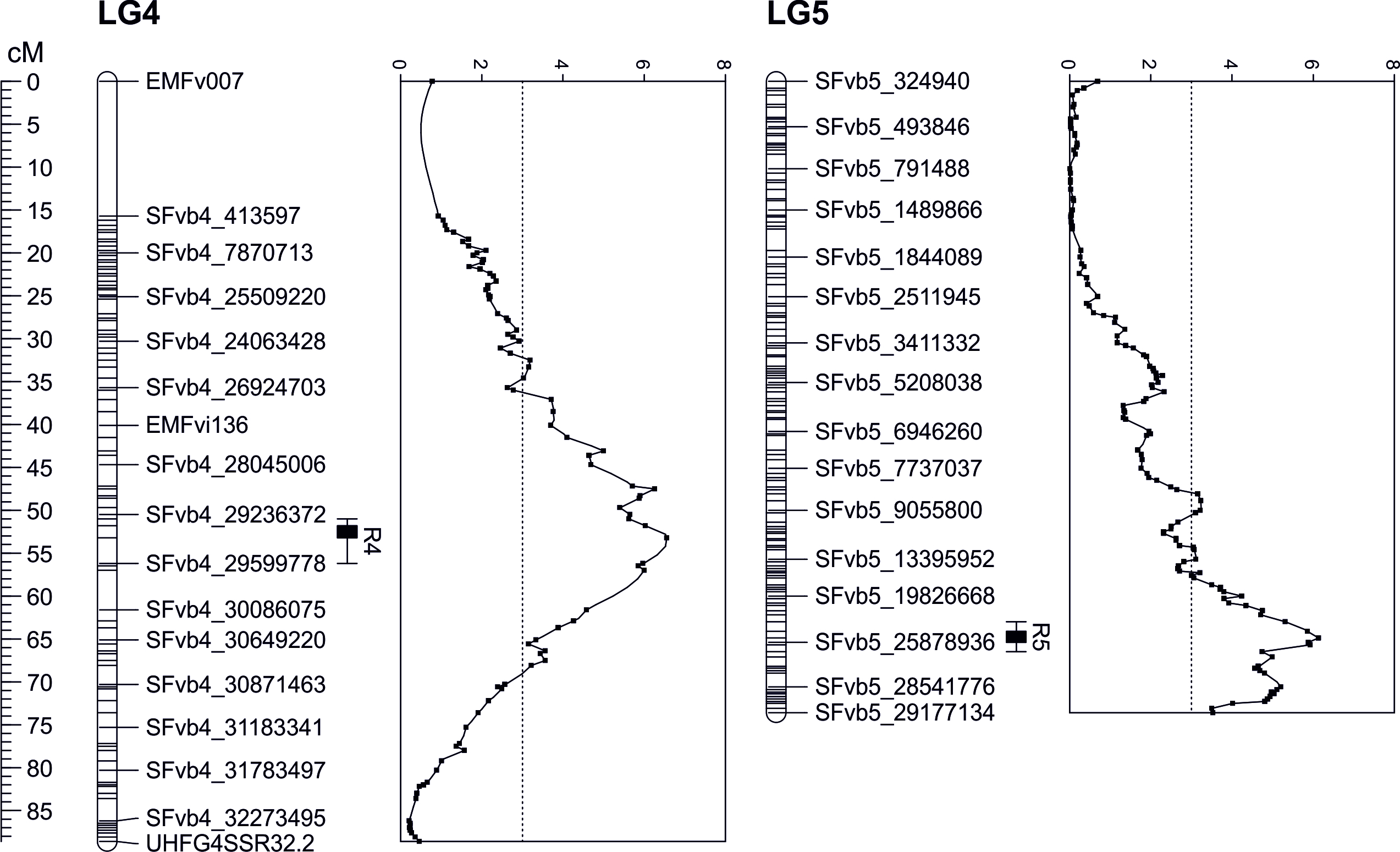

Supplement: Supplementary Figure 5 [file hortres201720-s6.jpg]
